# Factors influencing accelerated aging in patients with type 2 diabetes mellitus and coronary heart disease

**DOI:** 10.3389/fendo.2024.1416234

**Published:** 2024-07-31

**Authors:** Zehua Huang, Nana Liu, Shiyi Chen, Zhiren Chen, Peian Wang

**Affiliations:** Xuzhou Central Hospital, Affiliated Xuzhou Clinical College of Xuzhou Medical University, Xuzhou, Jiangsu, China

**Keywords:** type 2 diabetes mellitus, coronary artery disease, phenotypic age acceleration, influencing factors, ADA, TyG

## Abstract

**Objective:**

To investigate the factors influencing accelerated aging in patients with type 2 diabetes mellitus (T2DM) and coronary heart disease (CHD).

**Methods:**

A total of 216 patients diagnosed with T2DM and CHD between August 2019 and August 2023 at Xuzhou Central Hospital were selected. Patients were divided into an aging group and a non-aging group, based on the positive or negative values of phenotypic age acceleration (PhenoAgeAccel). Logistic regression analysis was conducted. Variables that had a univariate analysis *P*< 0.05 were included in the multivariate analysis to identify factors influencing aging in patients with T2DM and CHD, and the area under the curve of the model was reported.

**Results:**

This study included 216 patients, with 89 in the accelerated aging group, and 127 in the non-accelerated aging group. The average age of patients was 70.40 (95% CI: 69.10-71.69) years, with 137 males (63.4%). Compared with the non-accelerated aging group, patients in the accelerated aging group were older, with a higher proportion of males, and a higher prevalence of hypertension, stable angina pectoris, and unstable angina pectoris. Multivariate Logistic regression analysis indicated that the absolute value of neutrophils (NEUT#), urea (UREA), adenosine deaminase (ADA), and the triglyceride-glucose index (TyG) were risk factors for accelerated aging, while cholinesterase (CHE) was a protective factor. For each unit increase in NEUT#, UREA, ADA, and TyG, the risk of aging increased by 64%, 48%, 10%, and 789%, respectively. The overall area under the receiver operating characteristic (ROC) curve of the model in the training set was 0.894, with a 95% confidence interval (CI) of 0.851-0.938.

**Conclusion:**

NEUT#, CHE, UREA, ADA, and TyG are predictors of accelerated aging in patients with T2DM and CHD, with the model showing favorable overall predictive performance.

## Introduction

Diabetes mellitus, as a metabolic disease, has garnered increasing attention worldwide due to its escalating prevalence, emerging as a global public health challenge according to the latest epidemiological data. This disease primarily comprises type 1 diabetes mellitus and type 2 diabetes mellitus (T2DM), with the latter accounting for the vast majority of cases (1). Simultaneously, the prevalence of T2DM combined with coronary heart disease (CHD) is also on the rise, imposing a severe impact on the health of patients (2). Studies have indicated that the risk of CHD is significantly increased in patients with T2DM, which may be closely related to metabolic disorders and the development of atherosclerosis associated with diabetes (3).

The impact of aging on diabetic patients is crucial. With advancing age, diabetic patients can face higher health risks and more complex challenges in disease management (4). Aged patients with diabetes often have multiple chronic diseases and complications, such as hypertension, hyperlipidemia, and cardiovascular diseases, which not only exacerbate the disease burden but also increase the complexity and risk of treatment (5). Phenotypic age acceleration is an important indicator for assessing the biological aging of patients with diabetes (6). that patients with diabetes often undergo an accelerated process of biological aging, as evidenced by cellular and tissue-level changes, along with the exacerbation of clinical phenotypes. For example, patients with diabetes are more prone to cardiovascular complications, neurological damage, renal failure, and other age-related pathologies, which may be closely related to inflammation, oxidative stress, and apoptosis triggered by diabetes (7).

Current research on accelerated aging in patients with diabetes is still in its nascent stages, focusing mainly on the identification of biomarkers, elucidation of molecular mechanisms, and exploration of intervention measures. The factors associated with accelerated aging in patients with T2DM combined with CHD are not yet clear (6–15). Therefore, this study aims to analyze the clinical manifestations and laboratory investigations, including biomarkers, of patients with T2DM and CHD, to investigate clinical indicators with predictive value for aging in these patients, in hopes of establishing a more comprehensive diagnostic, assessment, and therapeutic approaches for patients with T2DM and CHD.

## Data and methods

### Study population

This study screened 345 patients diagnosed with diabetes and CHD at Xuzhou Central Hospital from August 2019 to August 2023; among them, 216 patients had complete baseline and laboratory test data. Inclusion criteria: Patients aged ≥18 years; the discharge diagnosis in the medical record includes both T2DM and coronary artery atherosclerotic heart disease (or CHD). Exclusion criteria: Patients with other types of diabetes; patients with incomplete baseline data or laboratory biomarker data. This study was approved by the hospital’s ethics committee (Approval number: XZXY-LK-20240108-0010).

### Methods

Clinical data collection: Retrospective collection of the clinical data of the subjects to obtain comprehensive information about the study population. This information covers the following five aspects:

(1) Demographic characteristics: Gender, age, marital status, occupation.
(2) Hospitalization information: exemption number, hospital day, readmission.
(3) Cost information: costs covered by medicare, out-of-pocket expenses, inspection fees, western medicine expenses, traditional Chinese medicine expenses, and payment method.
(4) Diagnostic information: secondary diagnoses, surgery, comorbidities (hypertension, cerebral hemorrhage, myocardial infarction, renal insufficiency, cardiac insufficiency, cerebral infarction, hemiplegia, stable angina pectoris (SAP), unstable angina pectoris (UAP), Parkinson’s disease).
(5) Laboratory test values: wide range of indicators including hematology, blood biochemistry, and urine routine. Specific indicators include: mean corpuscular volume (MCV), red cell distribution width (RDWC), white blood cells (WBC), lymphocyte percentage (LYMPH%), albumin (ALB), glucose (GLU), creatinine (CREA), high sensitivity C-reactive protein (HSCRP), alkaline phosphatase (ALP), red blood cells (RBC), hemoglobin (HGB), hematocrit (HCT), mean corpuscular hemoglobin (MCH), mean corpuscular hemoglobin concentration (MCHC), platelets (PLT), absolute neutrophil count (NEUT#), absolute monocyte count (MONO#), absolute eosinophil count (EO#), absolute basophil count (BASO#), neutrophil percentage (NEUT%), monocyte percentage (MONO%), basophil percentage (BASO%), eosinophil percentage (EO%), aspartate aminotransferase (AST), alanine aminotransferase (ALT), gamma-glutamyltransferase (GGTr), uric acid (UA), total protein (TP), cholinesterase (CHE), direct bilirubin (DBIL), total bilirubin (TBIL), urea (UREA), globulin (GLO), albumin/globulin ratio (ALB/GLO), calcium (CA), sodium (NA), potassium (K), chloride (CL), phosphorus (P), prealbumin (PA), serum cystatin C (CYSC), adenosine deaminase (ADA), magnesium (MG), estimated glomerular filtration rate (eGFR), apolipoprotein A (APOA), apolipoprotein B (APOB), alpha-hydroxybutyrate dehydrogenase (HBDH), high-density lipoprotein cholesterol (HDLC), lactate dehydrogenase (LDH), low-density lipoprotein cholesterol (LDLC), lipoprotein(a) (LPA), total cholesterol (TCH), triglycerides (TG).

### Phenotypic age acceleration

PhenoAge was calculated based on the actual age and a linear combination of 9 biomarkers (ALB, GLU, CREA, LOG (HSCRP), LYMPH%, MCV, RDWC, ALP, WBC) (16). PhenoAge reveals an individual’s physiological status, but more crucial is the concept of phenotypic age acceleration (PhenoAgeAccel). PhenoAgeAccel represents the rate of aging considering the actual age, i.e., whether an individual physiologically appears older (positive value) or younger (negative value) than expected (7). This indicator allows us to more accurately understand an individual’s health status and biological age.

Formula for calculating PhenoAge:


$$\mathrm{Pheno}\;\mathrm{Age}\;=\;141.50225\;+\frac{\text{ln}\left(-0.00553\times \text{ln}\left(1-\text{Mortality risk}\right)\right)}{0.090165}$$

where,


$$\mathrm{Mortality}\;\mathrm{risk}\;=\;1\;-e^{-e^{xb}\left(\text{exp}\left(120\text{γ}\right)-1\right)/\text{γ}}$$

γ=0.0076927

$$\begin{array}{c} xb=\;-19.9067\;-\;0.0336\;\times \;\mathrm{ALB}\;\left(\text{g}/\text{L}\right)\;+\;0.0095\;\times \;\\ \mathrm{CREA}\;\left(µ\mathrm{mol}/\text{L}\right)\;+\;0.1953\;\times \\ \mathrm{GLU}\;\left(\mathrm{mmol}/\text{L}\right)\;+\;0.0954\;\times \;\log\;\left(\mathrm{HSCRP}\right)\;\left(\mathrm{mg}/\mathrm{dL}\right)\\ \;-\;0.0120\;\times \;\mathrm{LYMPH}\%\;+\;0.0268\;\times \\ \mathrm{MCV}\;\left(\mathrm{fL}\right)\;+\;0.3306\;\times \;\mathrm{RDWC}\;\left(\%\right)\;+\;0.0019\;\times \;\mathrm{ALP}\;\left(\text{U}/\text{L}\right)\;+\\ \;0.0554\;\times \;\mathrm{WBC}\;\left(1000\;\mathrm{cells}/µ\text{L}\right)\;+\;0.0804\;\times \;\mathrm{AGE}\;\left(\mathrm{years}\right) \end{array}$$

PhenoAgeAccel = PhenoAge – AGE

The triglyceride-glucose (TyG) index is identified as a novel surrogate marker for insulin resistance (IR), which is associated with the incidence of diabetes and cardiovascular diseases (17). Therefore, it is also considered as one of the observational indicators. The TyG index is computed based on fasting plasma glucose (FPG) and triglycerides (TG). The formula for calculating the TyG index is as follows (18):


$$\begin{array}{c} \text{T}\text{y}\text{G}\;\text{i}\text{n}\text{d}\text{e}\text{x}=\text{L}\text{n}[\text{f}\text{a}\text{s}\text{t}\text{i}\text{n}\text{g}\;\text{t}\text{r}\text{i}\text{g}\text{l}\text{y}\text{c}\text{e}\text{r}\text{i}\text{d}\text{e}\text{s}\;(\text{m}\text{g}/\text{d}\text{L})\\ \;\times \;\text{f}\text{a}\text{s}\text{t}\text{i}\text{n}\text{g}\;\text{g}\text{l}\text{u}\text{c}\text{o}\text{s}\text{e}\;(\text{m}\text{g}/\text{d}\text{L})/2] \end{array}$$

In this study, patients were grouped based on the results of PhenoAgeAccel, into an accelerated aging group (PhenoAgeAccel > 0) (n = 89) and a non-accelerated aging group (PhenoAgeAccel ≤ 0) (n = 127) for analysis.

### Statistical methods

SPSS V26.0 was used for statistical analysis of the data. In this study, all quantitative data underwent Shapiro-Wilk normality tests and tests for homogeneity of variances. Variables with a normal distribution are presented as $\bar{x}\pm s$ and analyzed using t-tests or F-tests; variables with a skewed distribution were represented by quartiles (P_25_, P_75_) and analyzed using rank-sum tests. Categorical variables were expressed as percentages (%) and analyzed using the chi-square test. Independent variables such as baseline data and biomarkers were first analyzed using univariate regression analysis in relation to PhenoAgeAccel. Multivariate Logistic regression analysis was then conducted to analyze factors influencing accelerated aging in patients, but only if the *P* value from the univariate analysis was less than 0.05. The overall area under the curve (AUC) value of the model and its 95% confidence interval (CI) were also reported. Statistical significance was defined at *P*< 0.05.

## Results

### Comparison of baseline data and laboratory test indicators

**Figure 1:**
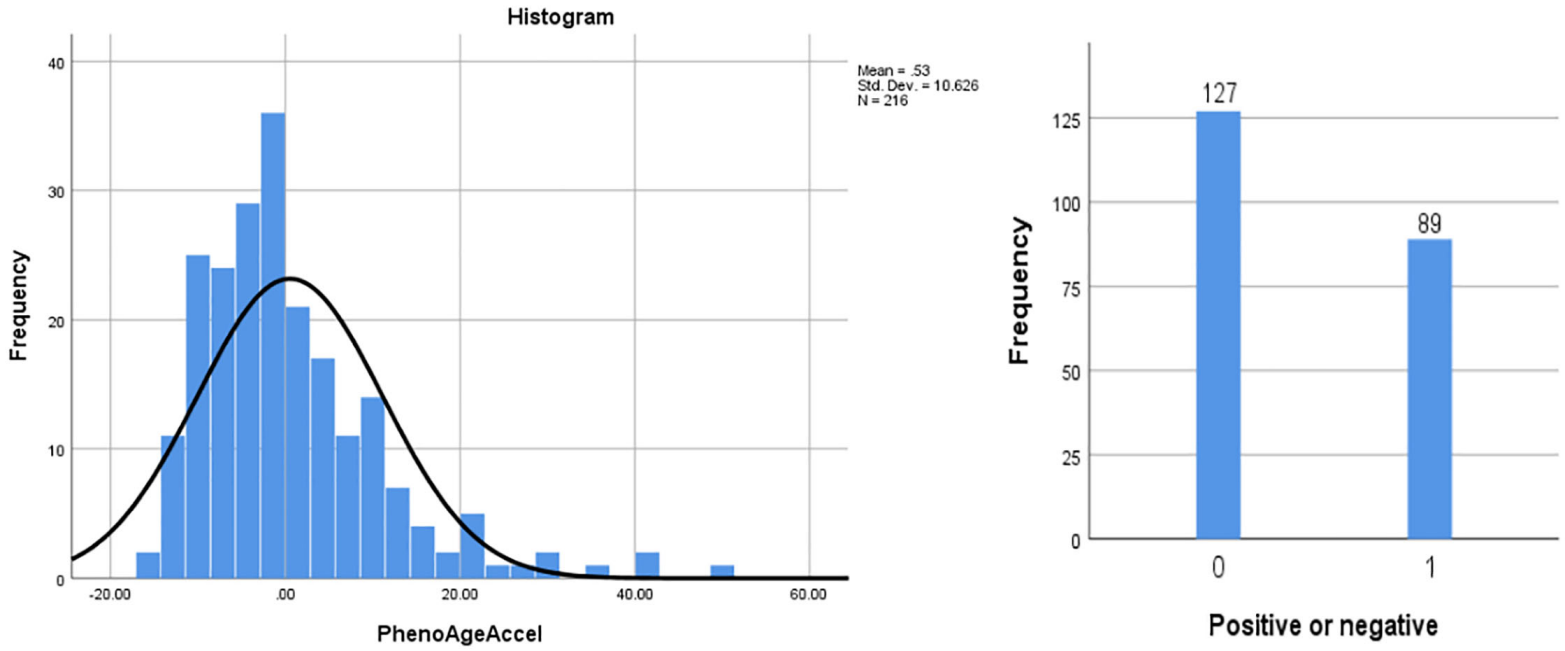
Histogram of PhenoAgeAccel.

The study included a total of 216 patients with T2DM and CHD, with an age of (70.40 ± 9.65) years; 79 females (36.6%) and 137 males (63.4%). The distribution of PhenoAgeAccel among patients was a skewed quantitative variable, with a mean of 0.529, a standard deviation of 10.626, and a quartile distribution (P_25_, P_75_) of (-6.799, 4.855). It was transformed into a categorical variable through grouping (**Figure 1**, **Table 1**).

**Table 1:** Descriptive statistical analysis of PhenoAge and PhenoAgeAccel.

statistic	PhenoAge	PhenoAgeAccel
N	216	216
Mean	70.925	0.529
SD	15.185	10.626
Min	30.368	-16.218
Max	133.285	50.187
Median	69.309	-1.704
P_25_	60.932	-6.799
P_75_	78.055	4.855
IQR	17.122	11.654
SE	1.033	0.723
95% CI	(68.88877, 72.96174)	(-0.89635, 1.95387)

Excluding the nine biomarkers that constitute PhenoAgeAccel, the differences between the accelerated aging group and the non-accelerated aging group were statistically significant (*P*< 0.05) in terms of TP, CHE, NEUT#, NEUT%, MONO#, UREA, NA, CL, PA, CYSC, MG, eGFR, APOA, HBDH, HDLC, TyG, renal insufficiency, and cardiac insufficiency. Compared with the non-accelerated aging group, patients in the accelerated aging group were older, with a higher proportion of males, and a higher prevalence of hypertension, stable angina pectoris, and unstable angina pectoris. Additionally, they undergo surgery more frequently, have a greater number of cumulative hospitalizations, and longer hospital stays. Moreover, their PLT, RDWC, WBC, GLU, CREA, HSCRP, ALP, NEUT#, MONO#, NEUT%, UA, DBIL, UREA, CYSC, ADA, APOB, HBDH, LDH, TG, and TyG values were higher (**Table 2**).

**Table 2:** Comparison of data between non-aging and aging groups in patients with T2DM and CHD.

Variable	Total (n = 216)	Positive or negative		P
		0 (n = 127)	1 (n = 89)	
AGE, Mean ± SD	70.40 ± 9.65	70.09 ± 8.95	70.83 ± 10.60	0.591
LYMPH%, Mean ± SD	25.79 ± 9.76	28.42 ± 8.52	22.03 ± 10.22	<.001
RBC, Mean ± SD	4.26 ± 0.63	4.33 ± 0.57	4.17 ± 0.71	0.056
PLT, Mean ± SD	209.36 ± 57.73	205.02 ± 55.65	215.54 ± 60.34	0.188
TP, Mean ± SD	67.61 ± 6.14	68.41 ± 6.00	66.48 ± 6.21	0.022
CHE, Mean ± SD	7525.91 ± 2092.55	7917.98 ± 1899.82	6966.43 ± 2234.23	<.001
CA, Mean ± SD	2.36 ± 0.13	2.36 ± 0.12	2.35 ± 0.14	0.404
P, Mean ± SD	1.13 ± 0.22	1.13 ± 0.21	1.13 ± 0.23	0.797
Exemption Number, M (Q_1_, Q_3_)	3.00 (1.00 - 5.00)	2.00 (1.00 - 5.00)	3.00 (1.00 - 6.00)	0.402
Hospital day, M (Q_1_, Q_3_)	10.00 (7.00 - 16.00)	9.00 (7.00 - 15.50)	11.00 (7.00 - 19.00)	0.265
Costs covered by Medicare, M (Q_1_, Q_3_)	11046.99 (5181.20 - 35081.94)	11399.40 (5245.37 - 36982.77)	10530.13 (5025.06 - 24729.38)	0.691
Out of pocket expense, M (Q_1_, Q_3_)	3322.43 (1878.32 - 9213.91)	3360.00 (1850.61 - 8673.24)	3316.75 (2000.00 - 9374.46)	0.796
Inspection fee, M (Q_1_, Q_3_)	2241.00 (1627.38 - 3790.18)	2208.00 (1653.00 - 3697.00)	2260.00 (1601.00 - 3939.00)	0.718
Western medicine expenses, M (Q_1_, Q_3_)	2571.44 (1190.49 - 6069.55)	2338.24 (1181.91 - 5524.06)	2799.67 (1202.81 - 7702.44)	0.264
Traditional Chinese medicine expenses, M (Q_1_, Q_3_)	797.50 (155.22 - 2093.72)	700.05 (149.76 - 1621.46)	991.43 (160.64 - 2444.98)	0.138
MCV, M (Q_1_, Q_3_)	91.15 (88.18 - 94.12)	91.90 (88.90 - 94.20)	90.90 (87.80 - 93.50)	0.437
RDWC, M (Q_1_, Q_3_)	13.00 (12.50 - 13.62)	12.90 (12.40 - 13.30)	13.20 (12.60 - 14.40)	<.001
WBC, M (Q_1_, Q_3_)	6.36 (5.48 - 7.70)	6.14 (5.26 - 7.14)	7.08 (5.81 - 9.03)	<.001
ALB, M (Q_1_, Q_3_)	41.00 (38.68 - 44.15)	42.00 (39.10 - 44.70)	40.00 (37.60 - 43.20)	0.009
GLU, M (Q_1_, Q_3_)	7.12 (5.62 - 9.00)	6.36 (5.29 - 7.55)	9.10 (6.98 - 11.65)	<.001
CREA, M (Q_1_, Q_3_)	62.50 (50.98 - 75.25)	57.90 (47.00 - 69.00)	68.80 (55.00 - 96.50)	<.001
HSCRP, M (Q_1_, Q_3_)	1.52 (0.50 - 6.31)	1.06 (0.50 - 3.30)	3.56 (0.87 - 17.77)	<.001
ALP, M (Q_1_, Q_3_)	74.00 (61.75 - 95.00)	71.00 (60.50 - 89.50)	81.00 (62.00 - 101.00)	0.022
HGB, M (Q_1_, Q_3_)	130.50 (116.00 - 143.00)	131.00 (119.50 - 144.50)	126.00 (111.00 - 141.00)	0.069
HCT, M (Q_1_, Q_3_)	39.10 (35.50 - 42.82)	39.50 (35.95 - 43.55)	38.10 (34.20 - 42.60)	0.072
MCH, M (Q_1_, Q_3_)	30.20 (29.20 - 31.22)	30.30 (29.20 - 31.30)	30.20 (29.20 - 31.20)	0.324
MCHC, M (Q_1_, Q_3_)	332.00 (322.00 - 338.00)	332.00 (325.00 - 338.00)	329.00 (321.00 - 339.00)	0.339
NEUT#, M (Q_1_, Q_3_)	4.00 (3.08 - 5.22)	3.61 (2.97 - 4.50)	4.60 (3.45 - 6.06)	<.001
MONO#, M (Q_1_, Q_3_)	0.52 (0.41 - 0.64)	0.49 (0.40 - 0.61)	0.54 (0.44 - 0.68)	0.025
EO#, M (Q_1_, Q_3_)	0.12 (0.06 - 0.20)	0.12 (0.06 - 0.18)	0.12 (0.06 - 0.23)	0.358
BASO#, M (Q_1_, Q_3_)	0.02 (0.01 - 0.04)	0.02 (0.01 - 0.03)	0.02 (0.01 - 0.04)	0.823
NEUT%, M (Q_1_, Q_3_)	61.95 (55.68 - 70.50)	59.40 (53.80 - 66.75)	66.70 (57.40 - 75.40)	<.001
MONO%, M (Q_1_, Q_3_)	8.00 (6.30 - 9.50)	8.00 (6.50 - 9.60)	7.50 (5.90 - 9.30)	0.169
BASO%, M (Q_1_, Q_3_)	0.40 (0.20 - 0.53)	0.40 (0.20 - 0.50)	0.40 (0.10 - 0.60)	0.543
EO%, M (Q_1_, Q_3_)	1.90 (0.97 - 3.00)	1.90 (1.05 - 3.00)	1.90 (0.90 - 3.10)	0.769
AST, M (Q_1_, Q_3_)	19.00 (15.88 - 25.00)	19.00 (16.00 - 25.00)	19.00 (15.00 - 25.00)	0.747
ALT, M (Q_1_, Q_3_)	19.60 (14.00 - 29.00)	21.00 (15.00 - 29.00)	18.00 (12.00 - 27.00)	0.130
GGTr, M (Q_1_, Q_3_)	23.00 (17.00 - 35.00)	23.00 (17.00 - 32.00)	23.00 (17.00 - 37.00)	0.346
UA, M (Q_1_, Q_3_)	306.50 (240.25 - 371.25)	291.00 (246.70 - 355.00)	312.00 (229.00 - 398.00)	0.220
DBIL, M (Q_1_, Q_3_)	3.80 (2.60 - 5.50)	3.80 (2.55 - 5.15)	4.00 (2.70 - 5.70)	0.402
TBIL, M (Q_1_, Q_3_)	10.75 (8.20 - 15.53)	11.00 (8.55 - 15.25)	10.40 (8.00 - 15.80)	0.554
UREA, M (Q_1_, Q_3_)	6.41 (5.12 - 8.16)	5.85 (4.74 - 7.06)	8.04 (5.85 - 10.41)	<.001
GLO, M (Q_1_, Q_3_)	26.10 (23.50 - 29.20)	26.20 (23.45 - 29.50)	26.10 (23.50 - 28.80)	0.896
ALB/GLO, M (Q_1_, Q_3_)	1.60 (1.40 - 1.80)	1.60 (1.40 - 1.80)	1.60 (1.30 - 1.70)	0.149
NA, M (Q_1_, Q_3_)	140.60 (138.47 - 142.70)	141.20 (139.10 - 143.00)	139.60 (137.60 - 141.60)	<.001
K, M (Q_1_, Q_3_)	3.95 (3.60 - 4.27)	3.97 (3.69 - 4.21)	3.92 (3.54 - 4.31)	0.848
CL, M (Q_1_, Q_3_)	101.95 (99.00 - 104.90)	102.30 (99.90 - 105.00)	101.00 (98.00 - 104.00)	0.031
PA, M (Q_1_, Q_3_)	229.20 (185.45 - 264.00)	239.50 (202.50 - 268.55)	216.00 (162.00 - 254.00)	0.004
CYSC, M (Q_1_, Q_3_)	1.03 (0.86 - 1.37)	0.98 (0.83 - 1.17)	1.18 (0.90 - 1.70)	<.001
ADA, M (Q_1_, Q_3_)	10.40 (7.68 - 14.50)	10.20 (7.60 - 13.60)	10.70 (7.90 - 16.10)	0.082
MG, M (Q_1_, Q_3_)	0.89 (0.81 - 0.93)	0.90 (0.84 - 0.96)	0.86 (0.79 - 0.91)	0.046
Egfr, M (Q_1_, Q_3_)	92.56 (80.60 - 100.45)	94.87 (86.81 - 101.68)	88.90 (59.80 - 97.28)	<.001
APOA, M (Q_1_, Q_3_)	1.19 (1.02 - 1.36)	1.24 (1.07 - 1.40)	1.10 (0.96 - 1.26)	<.001
APOB, M (Q_1_, Q_3_)	0.72 (0.60 - 0.90)	0.71 (0.60 - 0.92)	0.74 (0.57 - 0.88)	0.940
HBDH, M (Q_1_, Q_3_)	145.50 (132.00 - 169.00)	141.00 (128.00 - 159.30)	153.00 (135.00 - 176.00)	0.025
HDLC, M (Q_1_, Q_3_)	1.06 (0.89 - 1.27)	1.10 (0.92 - 1.33)	0.99 (0.83 - 1.18)	0.003
LDH, M (Q_1_, Q_3_)	190.00 (168.75 - 221.00)	189.00 (168.50 - 207.00)	194.00 (169.00 - 243.70)	0.102
LDLC, M (Q_1_, Q_3_)	2.25 (1.79 - 2.88)	2.26 (1.75 - 2.92)	2.21 (1.82 - 2.73)	0.674
LPA, M (Q_1_, Q_3_)	187.20 (85.75 - 374.62)	202.00 (89.05 - 369.50)	158.00 (79.00 - 396.30)	0.407
TCH, M (Q_1_, Q_3_)	3.81 (3.19 - 4.67)	3.97 (3.19 - 4.92)	3.72 (3.15 - 4.49)	0.290
TG, M (Q_1_, Q_3_)	1.24 (0.90 - 1.84)	1.19 (0.89 - 1.82)	1.36 (0.93 - 1.86)	0.406
Secondary diagnoses, M (Q_1_, Q_3_)	4.00 (3.00 - 6.00)	4.00 (3.00 - 6.00)	4.00 (3.00 - 6.00)	0.733
TyG, M (Q_1_, Q_3_)	8.91 (8.46 - 9.35)	8.68 (8.31 - 9.12)	9.05 (8.76 - 9.60)	<.001
Gender, n (%)				0.060
1	137 (63.43)	74 (58.27)	63 (70.79)	
2	79 (36.57)	53 (41.73)	26 (29.21)	
Payment Method, n (%)				0.564
1	142 (65.74)	86 (67.72)	56 (62.92)	
2	6 (2.78)	4 (3.15)	2 (2.25)	
3	46 (21.3)	28 (22.05)	18 (20.22)	
4	9 (4.17)	4 (3.15)	5 (5.62)	
5	4 (1.85)	1 (0.79)	3 (3.37)	
6	9 (4.17)	4 (3.15)	5 (5.62)	
Readmission, n (%)				0.879
0	57 (26.39)	34 (26.77)	23 (25.84)	
1	159 (73.61)	93 (73.23)	66 (74.16)	
Hypertension, n (%)				0.125
0	63 (29.17)	32 (25.20)	31 (34.83)	
1	153 (70.83)	95 (74.80)	58 (65.17)	
Cerebral hemorrhage, n (%)				0.764
0	209 (96.76)	122 (96.06)	87 (97.75)	
1	7 (3.24)	5 (3.94)	2 (2.25)	
Myocardial infarct, n (%)				0.073
0	198 (91.67)	120 (94.49)	78 (87.64)	
1	18 (8.33)	7 (5.51)	11 (12.36)	
Renal insufficiency, n (%)				0.026
0	206 (95.37)	125 (98.43)	81 (91.01)	
1	10 (4.63)	2 (1.57)	8 (8.99)	
Cardiac insufficiency, n (%)				0.001
0	183 (84.72)	116 (91.34)	67 (75.28)	
1	33 (15.28)	11 (8.66)	22 (24.72)	
Cerebral infarction, n (%)				0.937
0	139 (64.35)	82 (64.57)	57 (64.04)	
1	77 (35.65)	45 (35.43)	32 (35.96)	
Hemiplegia, n (%)				0.708
0	203 (93.98)	120 (94.49)	83 (93.26)	
1	13 (6.02)	7 (5.51)	6 (6.74)	
SAP, n (%)				0.385
0	213 (98.61)	124 (97.64)	89 (100.00)	
1	3 (1.39)	3 (2.36)	0 (0.00)	
UAP, n (%)				0.429
0	187 (86.57)	108 (85.04)	79 (88.76)	
1	29 (13.43)	19 (14.96)	10 (11.24)	
Parkinson, n (%)				0.135
0	213 (98.61)	127 (100.00)	86 (96.63)	
1	3 (1.39)	0 (0.00)	3 (3.37)	
Marital status, n (%)				0.143
1	211 (97.69)	126 (99.21)	85 (95.51)	
2	4 (1.85)	1 (0.79)	3 (3.37)	
3	1 (0.46)	0 (0.00)	1 (1.12)	
Occupation, n (%)				0.767
1	112 (51.85)	67 (52.76)	45 (50.56)	
2	33 (15.28)	20 (15.75)	13 (14.61)	
3	14 (6.48)	8 (6.30)	6 (6.74)	
4	4 (1.85)	2 (1.57)	2 (2.25)	
5	2 (0.93)	1 (0.79)	1 (1.12)	
6	1 (0.46)	0 (0.00)	1 (1.12)	
7	1 (0.46)	1 (0.79)	0 (0.00)	
8	1 (0.46)	0 (0.00)	1 (1.12)	
9	38 (17.59)	20 (15.75)	18 (20.22)	
10	10 (4.63)	8 (6.30)	2 (2.25)	
Surgery, n (%)				0.233
0	133 (61.57)	74 (58.27)	59 (66.29)	
1	83 (38.43)	53 (41.73)	30 (33.71)	
AGE group, n (%)				0.104
1	54 (25)	28 (22.05)	26 (29.21)	
2	54 (25)	34 (26.77)	20 (22.47)	
3	54 (25)	38 (29.92)	16 (17.98)	
4	54 (25)	27 (21.26)	27 (30.34)	

### Univariate and multivariate regression analysis on accelerated aging in patients with T2DM and CHD

Using accelerated aging as the dependent variable, a univariate Logistic regression analysis was conducted on patients with T2DM and CHD. The results indicated that there were statistically significant differences (*P*< 0.05) between the aging and non-aging groups in terms of HGB, HCT, NEUT#, NEUT%, TP, CHE, UREA, NA, PA, CYSC, ADA, eGFR, APOA, HBDH, HDLC, LDH, TyG, renal insufficiency, and cardiac insufficiency. After univariate regression analysis and limiting the independent variables with a *P* value of less than 0.05, these variables were subjected to a multivariate Logistic regression analysis. Multivariate analysis yielded significant results: NEUT# [odds ratio (OR) = 1.64; 95% CI: 1.21–2.23; *P* = 0.002], CHE [OR = 0.99; 95% CI: 0.99–0.99; *P* = 0.006], UREA [OR = 1.48; 95% CI: 1.16–1.88; *P* = 0.001], ADA [OR = 1.10; 95% CI: 1.02–1.19; *P* = 0.011], and TyG [OR = 8.89; 95% CI: 3.84–20.59; *P*< 0.001], were identified as influencing factors accelerating aging (**Table 3**).

**Table 3:** Univariate and multivariate regression analysis on accelerated aging in patients with T2DM and CHD.

Variables	OR (95%CI)	P	aOR (95%CI)	aP
Exemption Number	1.01 (0.97 - 1.05)	0.730		
AGE	1.01 (0.98 - 1.04)	0.578		
Hospital day	1.01 (0.98 - 1.04)	0.497		
Costs covered by Medicare	1.00 (1.00 - 1.00)	0.379		
Out of pocket expense	1.00 (1.00 - 1.00)	0.654		
Inspection fee	1.00 (1.00 - 1.00)	0.091		
Western medicine expenses	1.00 (1.00 - 1.00)	0.062		
Traditional Chinese medicine expenses	1.00 (1.00 - 1.00)	0.219		
RBC	0.65 (0.42 - 1.01)	0.058		
HGB	0.98 (0.97 - 0.99)	0.024	0.97 (0.88 - 1.07)	0.600
HCT	0.95 (0.90 - 0.99)	0.025	1.12 (0.79 - 1.58)	0.527
MCH	0.91 (0.78 - 1.06)	0.213		
MCHC	0.99 (0.96 - 1.01)	0.260		
PLT	1.00 (1.00 - 1.01)	0.188		
NEUT#	1.38 (1.18 - 1.62)	<.001	1.64 (1.21 - 2.23)	0.002
MONO#	3.36 (0.81 - 13.85)	0.094		
EO#	5.43 (0.83 - 35.43)	0.077		
BASO#	3359.01 (0.01 - 1823138447.17)	0.228		
NEUT%	1.06 (1.03 - 1.09)	<.001	0.99 (0.94 - 1.05)	0.738
MONO%	0.95 (0.85 - 1.05)	0.296		
BASO%	1.10 (0.42 - 2.88)	0.840		
EO%	1.06 (0.93 - 1.20)	0.408		
AST	1.01 (1.00 - 1.03)	0.089		
ALT	1.00 (0.99 - 1.01)	0.441		
GGTr	1.01 (1.00 - 1.01)	0.090		
UA	1.00 (1.00 - 1.01)	0.062		
TP	0.95 (0.91 - 0.99)	0.024	0.97 (0.89 - 1.06)	0.553
CHE	0.99 (0.99 - 0.99)	0.001	0.99 (0.99 - 0.99)	0.006
DBIL	1.05 (0.93 - 1.18)	0.412		
TBIL	1.01 (0.99 - 1.03)	0.428		
UREA	1.41 (1.22 - 1.62)	<.001	1.48 (1.16 - 1.88)	0.001
GLO	1.01 (0.95 - 1.07)	0.816		
ALB/GLO	0.44 (0.18 - 1.08)	0.072		
CA	0.40 (0.05 - 3.44)	0.403		
NA	0.89 (0.82 - 0.97)	0.008	1.03 (0.90 - 1.18)	0.706
K	1.06 (0.62 - 1.82)	0.818		
CL	0.95 (0.90 - 1.01)	0.125		
P	1.18 (0.34 - 4.15)	0.795		
PA	0.99 (0.99 - 0.99)	0.002	1.00 (0.99 - 1.01)	0.564
CYSC	5.55 (2.56 - 12.00)	<.001	3.10 (0.51 - 18.66)	0.217
ADA	1.07 (1.02 - 1.13)	0.008	1.10 (1.02 - 1.19)	0.014
MG	0.23 (0.03 - 1.92)	0.174		
Egfr	0.96 (0.95 - 0.98)	<.001	1.00 (0.97 - 1.04)	0.837
APOA	0.09 (0.02 - 0.29)	<.001	0.16 (0.01 - 2.18)	0.170
APOB	0.91 (0.31 - 2.66)	0.863		
HBDH	1.01 (1.01 - 1.02)	0.018	1.03 (1.00 - 1.06)	0.097
HDLC	0.21 (0.07 - 0.58)	0.003	1.72 (0.22 - 13.75)	0.607
LDH	1.01 (1.01 - 1.01)	0.019	0.98 (0.96 - 1.01)	0.146
LDLC	0.94 (0.69 - 1.27)	0.670		
LPA	1.00 (1.00 - 1.00)	0.989		
TCH	0.89 (0.70 - 1.12)	0.312		
TG	1.12 (0.89 - 1.42)	0.327		
Secondary diagnoses	1.03 (0.87 - 1.23)	0.727		
TyG	2.45 (1.60 - 3.76)	<.001	8.89 (3.84 - 20.59)	<.001
Gender				
1	1.00 (Reference)			
2	0.58 (0.32 - 1.03)	0.061		
Payment Method				
1	1.00 (Reference)			
5	4.61 (0.47 - 45.41)	0.191		
3	0.99 (0.50 - 1.95)	0.971		
6	1.92 (0.49 - 7.46)	0.346		
4	1.92 (0.49 - 7.46)	0.346		
2	0.77 (0.14 - 4.33)	0.765		
Readmission				
1	1.00 (Reference)			
0	0.95 (0.51 - 1.76)	0.879		
Hypertension				
1	1.00 (Reference)			
0	1.59 (0.88 - 2.87)	0.126		
Cerebral hemorrhage				
0	1.00 (Reference)			
1	0.56 (0.11 - 2.96)	0.496		
Myocardial infarct				
0	1.00 (Reference)			
1	2.42 (0.90 - 6.50)	0.080		
Renal insufficiency				
1	1.00 (Reference)		1.00 (Reference)	
0	0.16 (0.03 - 0.78)	0.023	1.43 (0.14 - 14.42)	0.759
Cardiac insufficiency				
1	1.00 (Reference)		1.00 (Reference)	
0	0.29 (0.13 - 0.63)	0.002	0.66 (0.21 - 2.10)	0.481
Cerebral infarction				
1	1.00 (Reference)			
0	0.98 (0.56 - 1.72)	0.937		
Hemiplegia				
0	1.00 (Reference)			
1	1.24 (0.40 - 3.82)	0.709		
SAP				
0	1.00 (Reference)			
1	0.00 (0.00 - Inf)	0.986		
UAP				
0	1.00 (Reference)			
1	0.72 (0.32 - 1.63)	0.431		
Parkinson				
0	1.00 (Reference)			
1	8502816.66 (0.00 - Inf)	0.985		
Marital status				
1	1.00 (Reference)			
3	3139890.32 (0.00 - Inf)	0.986		
2	4.45 (0.45 - 43.47)	0.200		
Occupation				
9	1.00 (Reference)			
1	0.75 (0.36 - 1.56)	0.438		
4	1.11 (0.14 - 8.72)	0.920		
2	0.72 (0.28 - 1.86)	0.500		
3	0.83 (0.24 - 2.87)	0.772		
5	1.11 (0.06 - 19.09)	0.942		
8	6397569.84 (0.00 - Inf)	0.991		
10	0.28 (0.05 - 1.48)	0.134		
6	6397569.85 (0.00 - Inf)	0.991		
7	0.00 (0.00 - Inf)	0.992		
Surgery				
0	1.00 (Reference)			
1	0.71 (0.40 - 1.25)	0.233		

### Model construction and validation for accelerated aging in patients with T2DM and CHD

**Figure 2:**
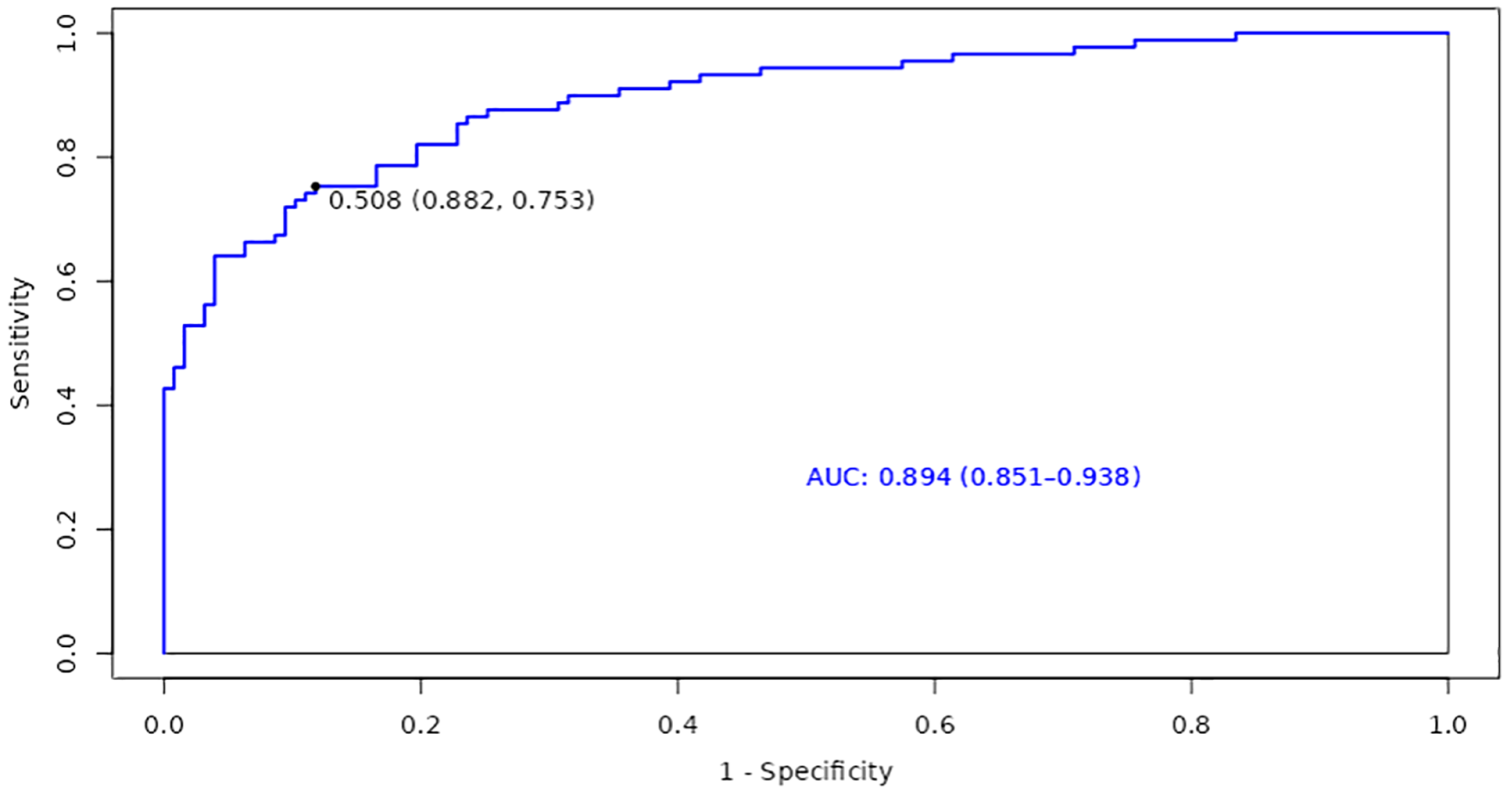
ROC Curve for the training set on accelerated aging in patients with T2DM and CHD.

Based on the identified factors related to accelerated aging in patients with T2DM and CHD, we focused on the construction and evaluation of a model to explore its performance on the training set, thereby gaining deeper insights into its predictive capability regarding whether patients are experiencing accelerated aging. Consequently, the AUC of the ROC curve for the training set, regarding whether patients with T2DM and CHD are experiencing accelerated aging, was 0.894 (95% CI: 0.851-0.938). This indicated that the model has a good discriminative ability in predicting whether patients are experiencing accelerated aging; the optimal cutoff value was 0.508, further establishing the accuracy of the prediction (**Figure 2**).

## Discussion

Based on a multivariate Logistic regression analysis, NEUT#, UREA, ADA, and TyG were deemed as risk factors for accelerated aging in patients with T2DM and CHD, while CHE was identified as a protective factor. Specifically, NEUT# was identified as a risk factor, with each unit increase escalating the risk of aging by 64%. Neutrophils serve as primary inflammatory mediators within the immune system, which typically increase in response to inflammation as part of the body’s immune response (19). In patients with T2DM and CHD, an elevated NEUT# may signify a persistent low-grade chronic inflammatory state, which has been shown to be closely associated with the development of diabetes (20). Neutrophils release a plethora of proinflammatory cytokines during the inflammatory process, such as tumor necrosis factor-alpha (TNF-)α and interleukin-6 (IL-6). These cytokines not only inflict direct damage on cardiovascular tissue but can also accelerate the aging process, making the active involvement of neutrophils a potential key driver of cardiovascular system aging in patients with T2DM (19). Furthermore, an increase in neutrophils may cause oxidative stress and inflammatory responses, culminating in impaired endothelial function, a seminal event in the pathogenesis of CHD (21). Previous studies have shown that neutrophils are related to the formation of atherosclerosis and plaque instability (22). Therefore, an increase in NEUT# may signify not only accelerated aging but also arterial pathology in patients with CHD. Studies have demonstrated that biomarkers, such as NEUT# and eGFR, are significantly associated with the risk of death in the aged inpatients affected by cardiovascular comorbidities (23).

CHE was identified as a protective factor in this study, with each unit increase reducing the risk of aging by 1%. CHE is an enzyme widely present in the human body, primarily tasked with hydrolyzing acetylcholine (ACh) into choline and acetic acid (24). ACh is a neurotransmitter that plays a key role in neuronal transmission, particularly at neuromuscular junctions and neuron-to-neuron synapses. When a nerve impulse is transmitted to the nerve terminal, ACh is released into the synaptic cleft, where it binds to receptors, initiates intracellular signal transduction, and induces corresponding physiological responses. Subsequently, it undergoes rapid hydrolysis by CHE, thereby terminating nerve transmission and averting excessive excitation (25). Besides its role in neuronal transmission, CHE also serves crucial biological functions in various tissues and organs. Within the cardiovascular system, cholinesterase plays a significant role, particularly in cardiac regulation. It influences cardiac rhythm and contractility by modulating the hydrolysis of ACh, thereby maintaining normal cardiac function (24). Research has found that the decrease in CHE activity may be related to chronic inflammation and oxidative stress (26). Chronic inflammation and oxidative stress are common features of many diseases, including cardiovascular diseases. In this case, a decrease in CHE activity could potentially affect the hydrolysis of ACh, and consequently influencing both neuronal transmission and cardiovascular function. Therefore, abnormalities in CHE activity may be one of the important factors influencing accelerated aging in patients with T2DM and CHD.

UREA was identified as a risk factor, with each unit increase escalating the risk of aging increases by 48%. UREA is one of the main metabolic waste products produced by protein metabolism, and its concentration is regulated by multiple factors, including renal function, protein metabolism rate, and water balance (27). With the presence of T2DM and CHD, changes in UREA level may signify abnormalities in the metabolic processes within the patient’s body Such abnormalities may implicate the patient’s renal function status, particularly regarding its role in the excretion of metabolic waste by the kidneys. Additionally, an increase in UREA level may be associated with the presence of chronic inflammation and oxidative stress within the patient’s body. These two physiological processes are notably important in the progression of cardiovascular aging. Chronic inflammation and oxidative stress can induce cellular aging and organ function degradation, thereby accelerating the progression of phenotypic aging. The changes in UREA concentration may also reflect the overall protein metabolism status of the patient (28). Protein metabolic disorder has been proven to be closely related to the development of diseases such as T2DM and CHD (29). Therefore, the abnormal changes in UREA level may not solely indicate renal function but also mirror shifts in the patient’s overall metabolic state, consequently impacting the occurrence of cardiovascular complications and hastening the process of phenotypic aging.

Furthermore, ADA was also a risk factor for accelerated aging in patients, with each unit increase escalating the risk of aging increases by 10%. ADA is crucial in the intracellular adenosine metabolism pathway, which catalyzes the conversion of adenosine to inosine. This reaction is essential for maintaining intracellular adenosine levels and regulating immune responses (30). Changes in ADA level within the patient’s body may signify the regulation of the immune system and inflammatory state, potentially impacting the acceleration of the phenotypic aging process. The activity of ADA is influenced by inflammatory responses, and its fluctuations may indicate the regulation of adenosine level by the inflammatory state, consequently impacting cellular metabolism and the aging process. Notably, in the cardiovascular system, the biological functions of adenosine are widely acknowledged to have a protective role. This protective effect encompasses, but is not limited to, the protection of vascular endothelial cells and the anti-ischemic effects during cardiac ischemia (31). Therefore, changes in ADA level within the patient’s body may be closely related to cardiovascular health, which further affects the function of the cardiovascular system, and thereby influencing the patient’s overall health and the development of phenotypic aging.

TyG was identified as a significant risk factor, with each unit increase escalating the aging risk for patients with T2DM and CHD by 789%. TyG is associated with insulin resistance, which is an important risk factor for T2DM and cardiovascular diseases (32). Insulin resistance can lead to metabolic abnormalities, including dyslipidemia and blood glucose increased, which are drivers of cardiovascular aging (33). Thus, the observed association between TyG and accelerated aging may partly reflect the mediating role of insulin resistance in cardiovascular aging. Furthermore, the elevation of TyG is closely related to increased lipid levels in patients. Triglycerides constitute a component of TyG calculation, and elevated blood lipid level is associated with the occurrence of atherosclerosis and cardiovascular events. Therefore, changes in TyG may affect the progression of cardiovascular aging by influencing lipid metabolism (34). TyG, as an easily calculable and widely applied index, has been proven to be closely related to cardiovascular risk in studies (17). The observed association between TyG and accelerated aging further emphasizes the potential role of TyG in predicting cardiovascular aging, which may offer a simple yet effective tool for early intervention and treatment.

Certain limitations are present in this study. Firstly, as an observational retrospective cohort study, it fails to elucidate the causal relationship between NEUT#, CHE, UREA, ADA, TyG, and the accelerated aging of patients with T2DM combined with CHD. Secondly, the study’s patients come from a single center with T2DM and coronary atherosclerotic heart disease, and the relatively small sample size cannot represent populations from other regions or of other ethnicities. Lastly, the cross-sectional nature of this study did not assess the predictive value of NEUT#, CHE, UREA, ADA, TyG for the outcome of accelerated aging in patients with T2DM and CHD, and long-term follow-up is still needed for further exploration. Despite these limitations, this study provides new biomarkers for the accelerated aging of T2DM and CHD patients, offering a novel perspective for clinical assessment. While this study has shed light on new biomarkers for accelerated aging in patients with T2DM and CHD, an integrated metabolomics analysis would deepen our understanding of the molecular changes during the aging process. Moreover, the supplementation of metabolomics is not only conducive to identifying new biomarkers associated with aging but also holds potential scientific value for the development of early diagnostic and personalized treatment strategies. Therefore, future research should consider incorporating metabolomics analysis into the framework of aging assessment to achieve a more comprehensive understanding of the aging process in patients with T2DM and CHD. In summary, after adjusting for confounding factors, NEUT#, CHE, UREA, ADA, and TyG have been identified as influential factors in the accelerated aging of patients with T2DM and CHD and may serve as economical and easily measurable laboratory indicators for predicting the biological aging condition in the future.

## Data availability statement

The datasets generated and/or analyzed during the current study are publicly available in the Figshare repository. The data can be accessed using the following DOI: 10.6084/m9.figshare.26198675.

## Ethics statement

The studies involving humans were approved by Biomedical Research Ethics Review Committee of Xuzhou Central Hospital committee (Approval number: XZXY-LK-20240108-0010). The studies were conducted in accordance with the local legislation and institutional requirements. Written informed consent for participation was not required from the participants or the participants' legal guardians/next of kin because The ethics committee approved the exemption of informed consent.

## Author contributions

ZH: Writing – original draft, Visualization, Methodology, Investigation, Formal analysis, Data curation, Conceptualization. NL: Writing – review & editing, Formal analysis, Data curation. SC: Writing – review & editing, Formal analysis, Data curation. ZC: Writing – review & editing, Formal analysis, Data curation. PW: Writing – review & editing, Validation, Supervision, Software, Resources, Project administration, Funding acquisition.
